# Organotypic 3D Co-Culture of Human Pleura as a Novel In Vitro Model of *Staphylococcus aureus* Infection and Biofilm Development

**DOI:** 10.3390/bioengineering10050537

**Published:** 2023-04-27

**Authors:** Olga Kurow, Rima Nuwayhid, Peggy Stock, Matthias Steinert, Stefan Langer, Sebastian Krämer, Isabella B. Metelmann

**Affiliations:** 1 Department of Orthopedic, Trauma and Plastic Surgery, University Hospital of Leipzig, 04103 Leipzig, Germany; 2 Department of Visceral, Transplant, Thoracic and Vascular Surgery, University Hospital of Leipzig, 04103 Leipzig, Germany; peggy.stock@medizin.uni-leipzig.de (P.S.); isabella.metelmann@medizin.uni-leipzig.de (I.B.M.)

**Keywords:** pleura, mesothelium, 3D co-culture, organotypic model, tissue engineering, *Staphylococcus aureus*, biofilm, antiseptic solutions therapy

## Abstract

Bacterial pleural infections are associated with high mortality. Treatment is complicated due to biofilm formation. A common causative pathogen is *Staphylococcus aureus* (*S. aureus*). Since it is distinctly human-specific, rodent models do not provide adequate conditions for research. The purpose of this study was to examine the effects of *S. aureus* infection on human pleural mesothelial cells using a recently established 3D organotypic co-culture model of pleura derived from human specimens. After infection of our model with *S. aureus*, samples were harvested at defined time points. Histological analysis and immunostaining for tight junction proteins (c-Jun, VE-cadherin, and ZO-1) were performed, demonstrating changes comparable to in vivo empyema. The measurement of secreted cytokine levels (TNF-α, MCP-1, and IL-1β) proved host–pathogen interactions in our model. Similarly, mesothelial cells produced VEGF on in vivo levels. These findings were contrasted by vital, unimpaired cells in a sterile control model. We were able to establish a 3D organotypic in vitro co-culture model of human pleura infected with *S. aureus* resulting in the formation of biofilm, including host–pathogen interactions. This novel model could be a useful microenvironment tool for in vitro studies on biofilm in pleural empyema.

## 1. Introduction

Bacterial pleura infections are a considerable healthcare challenge, and pleural empyema is still associated with high morbidity and mortality [1,2,3]. Pleural empyema is a purulent infection characterized by an uncontrolled and excessive growth of bacterial pathogens in patients’ pleural cavity, resulting in the accumulation of purulent exudate in the cavum pleurae. The main pathogenic bacteria in pleural empyema are *Streptococcus pneumoniae*, *Staphylococcus aureus* (*S. aureus*), and *Pseudomonas* spp. [1]. A severe aggravation and one of the factors leading to the lack of treatment progress may be that certain bacteria are able to encase themselves in a protective coating, organizing in so-called biofilms [3]. 

Biofilms develop over hours and days from planktonic bacteria (single-celled). One advantage of biofilm growth over planktonic growth is the increased protection against antibiotics, phagocytes, antibodies, bactericides, and other anti-infectives [4,5]. The tolerance of *S. aureus* in biofilm towards antibiotics is up to a hundred times higher compared to planktonic cells [6]. The presence of extracellular substances (virulence factors) inside the biofilm also increases resistance to antibiotics and the immune system response [7].

Multiple virulence factors of *S. aureus*, which inhibit complement activation, block, or even destroy phagocytic cells, have unique human tropisms. Hence, *S. aureus* is a human-specific pathogen and cannot be studied adequately in rodents. Nevertheless, until now, mice were the most commonly chosen experimental animals for modeling human *S. aureus* infections. Due to significant histological and immunological differences between humans and rodents, there is increasing evidence that preclinical rodent models mimic the disease poorly [8,9,10]. Therefore, a realistic in vitro cell culture model is required for studying *S. aureus* infections.

Two-dimensional models such as monolayer or multilayer cultures are still predominantly used for in vitro drug testing, despite their insufficient representation of physiological conditions [11]. Among their numerous limitations are the restriction to one cell type (monolayer cultures), lack of tissue-typical architecture and extracellular matrix, and marginal control over cell type composition [12]. While spheroid models comprise multiple cell types in a 3D structure, this structure is confined to cell clusters instead of an organotypic layered architecture [13]. 

Three-dimensional (3D) in vitro organoids represent an established, cost-effective approach to model organs or tissues and to investigate pathogenesis without animal experiments [14,15,16].

Several protocols to grow organoids from adult human tissues and cell lines have been described, such as those derived from the colon, heart, intestine, liver, pancreas, stomach, esophagus, prostate, lung, breast, and fallopian tube, and from various cancers [17,18,19,20,21,22,23]. Tumor organoids have previously been shown to phenocopy their tumor of origin, allowing in vitro drug responses to be linked to genetic alterations present in the original tumor [24].

We introduced a 3D organotypic co-culture model of pleura to imitate the distinct pleural microenvironment. This mesothelial 3D model is derived from human pleura specimens and effectively reproduces the in vivo morphology as well as the microenvironment of human pleura [25]. Thus, it serves as an in vitro assay for pleural research. 

This study aims to mimic the evolution of a bacterial biofilm of *S. aureus* and investigate its influence on pleural mesothelial cells. Our 3D co-culture model of pleura is a novel tool, which can be used inter alia for in vitro studies to evaluate and test medical products and therapeutics. 

## 2. Materials and Methods

### 2.1. Three-Dimensional Organotypic Co-Culture Model of Pleura

**Figure 1:**
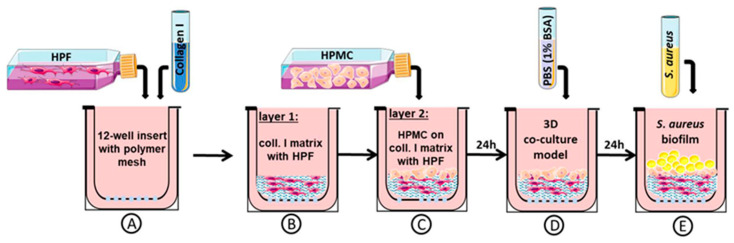
Schematic protocol for building a 3D organotypic model of pleura and its infection with bacteria. HPFs were mixed with collagen type I (**A**) to generate layer 1 (**B**). Layer 1 was polymerized and HPMCs were placed on top to build layer 2, solidifying for 24 h (**C**). Then, 24 h before bacterial inoculation, the medium was changed to PBS with BSA (1%) without antibiotics (**D**). Infection of 3D co-culture model with *S. aureus* was induced (**E**).

The 3D organotypic co-culture model of pleura was set up with human pleura mesothelial cells (HPMCs) and human pleural fibroblasts (HPFs) (Figure 1A–C), which were obtained from patients undergoing thoracic surgery at the University Hospital of Leipzig, University of Leipzig, Medical Center, Germany, Department of Thoracic Surgery (approved by the ethics committee of the University of Leipzig (477/20-ek)).

Human pleura biopsy specimens were crushed into small segments and disaggregated with trypsin (phosphate-buffered saline (PBS) and 0.25% trypsin/25 mM ethylendiamintetraacetat (EDTA) (1:2, *v*/*v*) for 40 min in order to isolate HPMCs. For isolation of HPFs, the trypsin/EDTA digestion (5 mL trypsin/EDTA/glucose (0.125%/0.01%/0.1 wt/vol in PBS)) was repeated twice for 20 min each at 37 °C in an orbital shaker with samples from the same biopsy. The cells obtained during each disaggregation step were collected in 25 cm^2^ culture flasks with 5 mL of Roswell Park Memorial Institute (RPMI) 1640 medium, 20% fetal bovine serum (FBS), and 100 U/mL penicillin, as well as 100 µg/mL streptomycin. The protocol for isolation of primary cells was previously described in detail by Metelmann et al. [25]. The primary cultures were incubated at 37 °C and 5% CO_2_ in 5 mL of RPMI 1640, 20% FBS, and 100 U/mL penicillin/100 µg/mL streptomycin in 25 cm^2^ culture flasks for 7 days with medium changed every 72 h. Isolated HPMCs and HPFs were purificated by immunofluorescence staining (previously described in detail by Metelmann et al. [25]). 

HPMCs and HPFs from the same pleura biopsy were used for the construction of 3D organotypic co-cultures (Figure 1). First, 5 × 10^4^ HPFs (per transwell) were mixed with 1 vol of acid-soluble rat tail collagen high concentration (HC), Type I (ibidi, Gräfelfing, Germany), 8 vol of Dulbecco’s modified eagle medium (DMEM) (DMEM, 10% FBS, and 100 U/mL penicillin/100 lg/mL streptomycin), and with 1 vol of NaOH buffer (0.1 N NaOH) (Figure 1A). Gel solidification took place in 2 h at 37 °C and 5% CO_2._ To build the collagen matrix with fibroblasts = layer 1 (Figure 1B), layer 1 was coated with 100 µL fibronectin (5 mg/mL). HPMCs (3.5 × 10^5^ per transwell) were then placed on top to build layer 2 (Figure 1C). An amount of 1 mL of growth medium (RPMI 1640, 20% FBS, and 100 U/mL penicillin/100 µg/mL streptomycin) was added inside and outside the insert. We allowed further incubation at 37 °C and 5% CO_2_ for 24 h to form a 3D co-culture model of pleura (Figure 1D).

One day before bacterial exposure of the co-culture model, the culture medium (RPMI 1640, 20% FBS, and 100 U/mL penicillin/100 µg/mL streptomycin) was switched to PBS with 1% bovine serum albumin (BSA) without antibiotics (Figure 1D). All experiments were performed on day one co-cultures. To induce infection, the apical side of the 3D organotypic co-culture model of pleura was exposed to bacterial culture (Figure 1E). Afterwards, samples from the co-culture were frozen or directly used in experiments.

### 2.2. Bacterial Strain

*S. aureus* strain ATCC 49230 was used because of previous experience and the virulence factors described [26]. This strain was obtained from the Institute for Microbiology of the University Hospital Leipzig. The strain was grown at 37 °C in casein-peptone-soy meal-peptone broth (Merck KGaA, Darmstadt, Germany) until the late exponential growth phase and stored at −80 °C in 20% sterile glycerol until usage for assays. For the experimental infection of the 3D co-culture model, an overnight plate culture of *S. aureus* was resuspended in 5 mL of casein-peptone-soy meal-peptone broth. This suspension with a turbidity equivalent to McFarland Standard no. 1, corresponding to 3 × 10^8^ colony-forming units (CFUs), was then diluted to a final concentration of approximately 10^3^–10^6^ CFU/mL.

### 2.3. Three-Dimensional Organotypic Co-Culture Model of Pleura and Bacterial Infection

To induce an infection, 20 μL of a bacterial suspension (10^3^ to 10^6^ CFU/mL) was transferred onto the apical side of the 3D co-culture model. This bacterial concentration is based on our previous infection studies, which we carried out on mice [26,27], as well as the concentration used in the studies by Charles et al. on tissue-engineered skin to study in vitro biofilm development [17]. As shown in Figure 1C, 24 h before bacterial inoculation of the co-culture model, the medium was changed to PBS with BSA (1%) (Sigma Aldrich, Taufkirchen, Germany) without antibiotics. The 3D co-cultures were treated with saline as a control or infected with biofilm-forming *Staphylococcus*. After further cultivation, samples of the surrounding medium and co-cultivated cells were harvested for analysis 3 h, 6 h, 12 h, and up to one day after infection or stored at −80 °C for further investigations. The experimental design of the 3D co-culture model infection is shown schematically in Figure 1.

### 2.4. Histological Analysis and Immunostaining

For histological analysis, the samples were placed in zinc-formaldehyde (Sigma Aldrich, Taufkirchen, Germany), embedded in paraffin, and cut into 7 µm sections. Standard hematoxylin and eosin (H&E, Carl Roth, Karlsruhe, Germany) and immunofluorescence staining were performed.

Histological severity scoring was performed in a double-blinded manner using the following criteria: 0—unaffected co-culture, 1—mild injury with minor mesothelial loosening, 2—moderate injury with some mesothelial disruption, 3—severe injury with continuous mesothelial disruption and some detachment, and 4—extensive injury, massive mesothelial disruption and detachment. 

For immunofluorescence staining, sections were stained as previously described [25] with primary antibodies against cellular Jun (c-Jun) (Cell Signaling, Danvers, MA, USA) diluted in blocking buffer (1:100), vascular endothelial cadherin (VE-cadherin) (1:250), as well as zonula occludens-1 (ZO-1) (1:1000) (Proteintech, Rosemont, IL, USA). After three washes in tris-buffered saline (TBS) for 10 min, slides were incubated with goat-anti rabbit Alexa Fluor-labeled secondary antibodies (Cell Signaling, Danvers, MA, USA) diluted in blocking buffer.

### 2.5. Cytokine and Growth Factor Quantification

An infected mesothelium produces inflammatory cytokines, such as tumor necrosis factor-α (TNF-α), interleukin-1β (IL-1β), monocyte chemoattractant protein-1 (MCP-1), and others. Cell-free supernatants were processed for cytokine secretion by enzyme-linked immunoabsorbent assay (ELISA) kits: vascular endothelial growth factor (VEGF), TNF-α, MCP-1, and IL-1β (R&D Systems, Minneapolis, MN, USA). Absorbance was measured at λ450/540 nm with SpectraMax 190 ELISA-Reader and analyzed with Softmax Pro Version 5.0 (Molecular Devices, San José, CA, USA) software. All samples were analyzed twice.

### 2.6. Barrier Integrity Assessment

*S. aureus* infection of pleura is determined by changes in the mesothelium itself, tissue integrity, and tissue permeability. To evaluate the barrier integrity, the permeability of the 3D co-culture model of pleura was assessed as previously described [25]: On day 1, after gel construction, the bacterial suspension (10^6^ CFU/mL) or serum-free medium (SFM) was pipetted onto the apical side of the 3D co-culture model. After further cultivation of the 3D co-culture model of pleura with bacteria for 3 h, 6 h, 12 h, and 24 h, the fluorescein-5-isothiocyanate (FITC) assay (Sigma-Aldrich, Saint Louis, MO, USA) was performed as previously described [25].

### 2.7. Determination of Cell Viability

To ensure that the bacterial infection reflected a clinically relevant scenario, cell viability was assessed using a Cytotoxicity Detection Kit (Roche Diagnostics GmbH, Mannheim, Germany) according to the manufacturer’s protocol. Absorbance was measured at λ490 nm with SpectraMax 190 ELISA-Reader and analyzed with Softmax Pro Version 5.0 (Molecular Devices, San José, CA, USA). All samples were analyzed in triplicate.

### 2.8. Characterization of Biofilm in the 3D Co-Culture Model of Pleura

To determine the number of viable cells by plate count (CFU), the non-adherent bacteria were removed by aspiration and each sample was cut into two equally sized pieces. One piece was used for histology and the other was transferred to 1 mL PBS (pH 7.4) and vortexed for 1 min to homogenize the biofilm. Next, the bacterial suspension was diluted and 100 µL of each dilution was plated on Mueller–Hinton agar (Carl Roth, Karlsruhe, Germany), followed by incubation overnight at 37 °C. Colonies were then counted and final counts calculated, taking the dilution factor into consideration.

### 2.9. Quantification of Penetration and Bacterial Accumulation

Light microscopy for biofilm characterization in the 3D co-culture model of pleura can be used to quantify localization (depth of layer penetration), accumulation, and formation of bacteria. For localization, bacteria in deeper regions (layer 1, collagen matrix; Figure 1B) were considered as a more severe infection than within the upper layer (layer 2, mesothelial cell layer; Figure 1C). The formation of bacteria was classified in descending degree of severity as mature biofilm, biofilm, bacterial aggregates, micro-colonies, single bacteria cells, and no bacteria. 

The colony-forming unit assay was performed to assess the transmigration of *S. aureus* across the mesothelial monolayer after different infection time points. Therefore, 100 μL of medium from the bottom compartment was collected and directly plated onto Mueller–Hinton agar plates. Plates were incubated overnight at 37 °C and colonies were then counted.

### 2.10. Statistical Analysis

Differences between groups by severity score and ELISA were evaluated by an unpaired two tailed *t*-test. For cytotoxicity experiments, differences were evaluated using two 2 × 2 ANOVA, each incorporating time as a within-subject characteristic (difference between first measurement and the last measurement at 24 h post infection). The data were analyzed using the GraphPad Prism v.5 software (GraphPad, San Diego, CA, USA). Differences were considered significant with *p* < 0.05.

## 3. Results

We engineered, validated, and tested a 3D organotypic model of human pleura previously described [25] in order to study the biofilm development of *S. aureus* strain ATCC 49230.

### 3.1. Morphology of 3D Organotypic Co-Culture Model of Pleura after Infection

**Figure 2:**
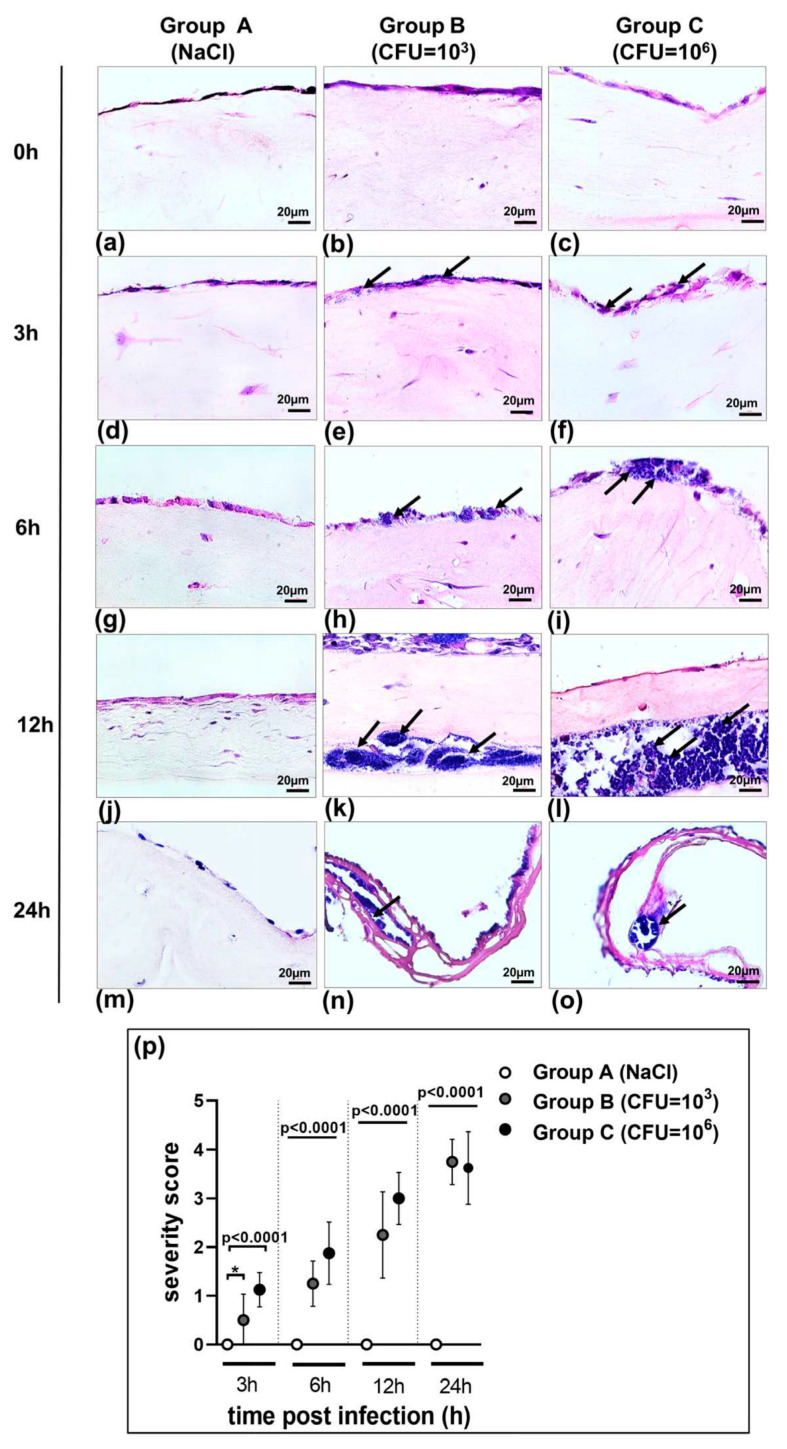
(**a**–**o**) Application of the 3D co-culture model of pleura in studying host–pathogen interactions. The organotypic co-culture model of pleura was infected with *S. aureus* strain ATCC 49230 for 3 h, 6 h, 12 h, or 24 h with 1 × 10^3^ and 1 × 10^6^ CFU/mL and tissue damage was analyzed by hematoxylin and eosin staining (**a**–**o**). In infection group B we detected bacterial accumulation on the mesothelial cell layer at 6 h post infection (**h**) and bacteria cells infiltrating soft tissue with formation of mature biofilm under the mesothelial layer (arrows) (**k**). In infection group C, the accumulation of bacteria on mesothelial cells was more significant than that in group B (**i**). This was accompanied by a large amount of bacterial cell infiltration and showed numerous, highly active cocci in biofilm formation distributed in the tissue (arrows) (**l**). Bacterial invasion of the tissue and biofilm building were consistent with tissue damage and capsule formation (arrows) (**n**,**o**) after 24 h exposure to *S. aureus* in both groups without a difference (B and C). Few inflammatory cells infiltrated the pleura in the control group (A). The saline control group (A) was aseptic. Images are from a single experiment and are representative of each group. Scale bar = 20 µm. (**p**) Histological severity scoring of tissue pathology of the 3D co-culture model of pleura exposed to *S. aureus* strain ATCC 49230. Histological severity scoring was performed in a double-blinded manner using the following criteria: 0, unaffected tissue; 1, mild injury with minor mesothelial loosening; 2, moderate injury with some mesothelial disruption; 3, severe injury with continuous mesothelial disruption and some detachment; 4, extensive injury, massive mesothelial disruption, and detachment (n = 8). The bars show the mean ± s.d. of two individual experiments. * *p* = 0.0192.

To ensure that the stimulation of bacterial infection on the 3D organotypic model of pleura reflected a clinically relevant scenario, our model was treated with saline as a control (Figure 2, group A) and infected with the biofilm-forming *Staphylococcus* strain (ATCC 49230) in two different concentrations: group B, 10^3^ CFU cells/mL, and group C, 10^6^ CFU/mL.

As shown in Figure 2 (H&E staining of the 3D co-culture of pleura), sections of group A (control) cultured in a bacteria-free medium demonstrated a fully intact mesothelial and submesothelial connective layer construct up to 24 h after saline application.

Both bacterial concentrations (group B 10^3^ and group C 10^6^ CFU/mL) infected the co-culture. Three hours after infection, bacteria were localized on the surface of the mesothelial cells, with some already infecting the remaining cells in the higher concentrated group C (Figure 2e,f). Significant injury (*p* = 0.0192) with minor mesothelial loosening was detected in these groups compared to the control group at 3 h (Figure 2p). Groups B and C demonstrated greater dissemination at 6 h (Figure 2h,i) and an increased biofilm formation at 12 h post infection (Figure 2k,l). As a consequence of infection, severe damage was observed as early as 6 h and increased significantly (*p* < 0.0001) over time (Figure 2p). At 24 h post infection, bacteria in both stimulated groups B and C were disseminated throughout the whole model and induced severe injury, disruption of the submesothelial connective layer, and capsule formation (Figure 2n,o).

### 3.2. Cytokine Production in 3D Organotypic Co-Culture Model of Pleura after Infection

**Figure 3:**
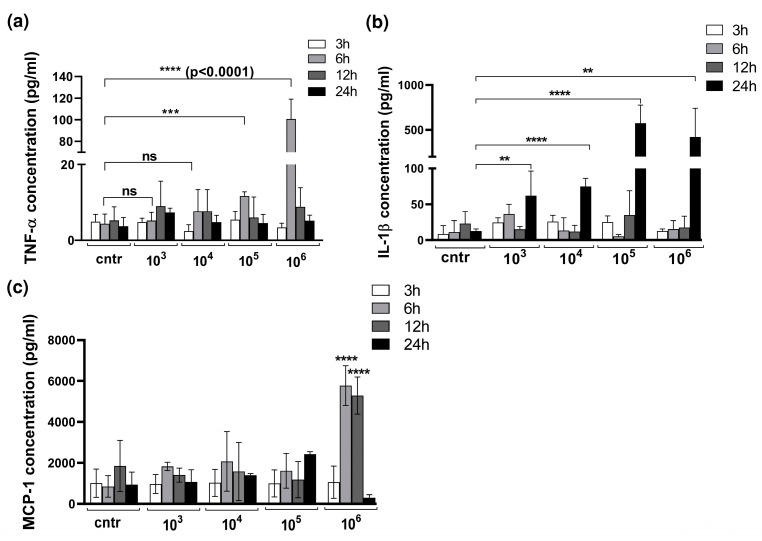
Infection affects cytokine production by 3D co-culture model of pleura. Measurement of TNFα (**a**), Il-1β (**b**), and MCP-1 (**c**) production in the 3D co-culture model of pleura upon infection with *S. aureus*. The medium was collected and the concentration measurements of TNFα, IL-1β, and MCP-1 were performed using ELISA. The graphs represent the mean values ± SD from at least three independent experiments. Statistical significance of cytokine concentration changes compared to non-infected control was tested by Student’s *t*-test: ** *p* = 0.0027, *** *p* ≤ 0.001, **** *p* ≤ 0.0001 ns = not significant.

Since bacterial infection of the mesothelium is characterized by a production of pro-inflammatory cytokines and an influx of innate immune cells to the site of infection, we assessed the levels of pro-inflammatory cytokines TNFα, IL-1β, and MCP-1 (Figure 3a,b).

We found significantly increased levels of TNF-α in models exposed to bacteria in higher CFUs, such as 10^5^ and 10^6^ CFU/mL (Figure 3a). Notably, models exposed to lower bacteria concentrations, such as 10^3^ and 10^4^ CFU/mL, elicited relatively mild damage and demonstrated a lower TNF-α response. In contrast, significantly increased levels of TNF-α were detected 6 h after stimulation with *S. aureus*, decreasing rapidly after that. 

The production of cytokine IL-1β showed no increase 3 h to 12 h after infection with *S. aureus* but rose abruptly at 24 h post infection (Figure 3b). 

The highest level of MCP-1 was measurable when the 3D model was infected with 10^6^ CFU/mL of *S. aureus*.

### 3.3. S. aureus Potently Induces Death of Pleural Fibroblasts and Mesothelial Cells Followed by Destruction of 3D Co-Culture Model of Pleura

**Figure 4:**
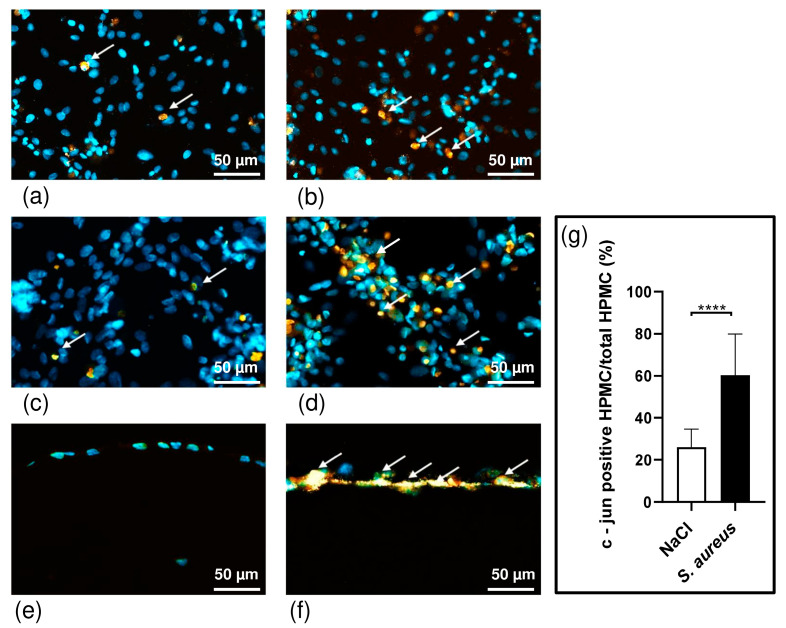
Immunohistochemically analysis of paraffin-embedded tissue. Immunohistochemistry staining of non-infected HPFs (**a**), HPMCs (**c**), and 3D co-culture model of pleura (**e**) in contrast to infected HPFs (**b**), HPMCs (**d**), and 3D model (**f**). Anti c-Jun (orange) and 4′,6-Diamidino-2-phenylindol (DAPI) (blue). Percentage of c-Jun-positive HPMCs of all cells within the 3D co-culture model. There are significantly more c-Jun-positive cells in the *S. aureus* infected group (indicated by arrows). The percentage of c-Jun-positive area is significantly higher in the *S. aureus* group (n = 11) than in the control group (n = 11); **** *p* ≤ 0.0001, unpaired *t*-test (**g**).

C-Jun plays an important role in controlling cellular proliferation, growth, and apoptosis. Increased c-Jun activity causes cell death in lung epithelial cells and fibroblasts [28,29]. HPMCs have been shown to express c-Jun in response to bacteria such as *S. aureus,* and levels of c-Jun are significantly increased in infectious pleural effusions [30]. *S. aureus* induces c-Jun, which may contribute to the activation of proapoptotic caspase-3, thereby resulting in apoptosis of PMCs during acute inflammation and empyema [31]. After infection of HPFs and HPMCs and the 3D co-culture with *S. aureus* (CFU 10^6^/mL) for 6 h, we performed immunohistochemistry staining with a c-Jun antibody (Figure 4a–f). The results of the immunohistochemistry showed that all samples, HPFs, HPMCs, and the 3D co-culture of both cell types produce c-Jun (Figure 4a–f).

We clearly registered more c-Jun signals (orange stain marked) in the infected co-culture compared to our non-infected co-culture serving as a control (*p* ≤ 0.0001) (Figure 4g).

In contrast to HPFs, we detected an increase in c-Jun activity in response to *S. aureus* infection by HPMCs after 6 h of incubating the cells with bacteria (Figure 4b,d). 

**Figure 5:**
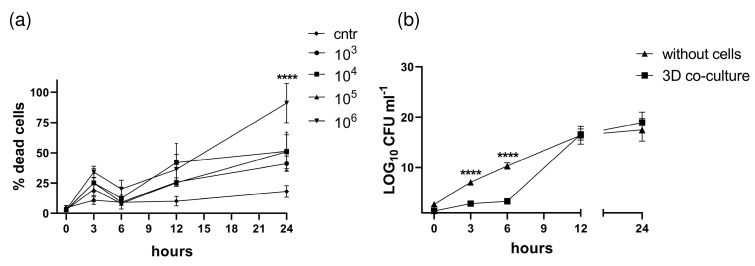
(**a**) Infection with *S. aureus* induces death of pleural mesothelial cells. The organotypic co-culture model of pleura was infected with *S. aureus* strain ATCC 49230 for 3 h, 6 h, 12 h, or 24 h with 1 × 10^3^ to 1 × 10^6^ CFU/mL. The cytotoxicity of *S. aureus* was evaluated using LDH assay. The graphs represent the mean values ± SD from at least three independent replicates. Statistical significance of LDH changes compared to non-infected control was tested by Student’s *t*-test: **** *p* ≤ 0.0001. (**b**) Bacterial transmigration assay. Bacterial transmigration through the transwell (no cells seeded) and 3D organotypic co-culture model of pleura. Transwell inserts or 3D co-culture models were infected with *S. aureus* (1 × 10^6^ CFU/mL) for 3 h, 6 h, 12 h, and 24 h. The number of bacteria in the basal compartment was calculated by plating and CFUs. All graphs represent the mean values ± SD from three independent experiments.

To investigate the direct cytotoxic effect of bacterial infection on pleural mesothelial cells and fibroblasts, the 3D co-culture model was infected with the clinical isolate *S. aureus* strain ATCC 49230, and lactate dehydrogenase (LDH) release was determined. The time-course effect of infection on 3D co-cultured cells over the 24 h treatment period is shown in Figure 5a.

**Figure 6:**
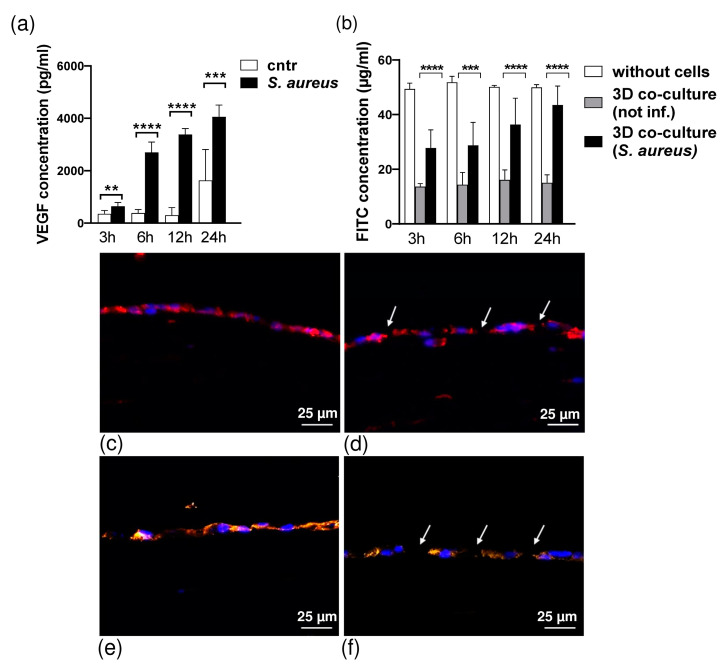
(**a**). *S. aureus* activated 3D co-culture model of pleura release of VEGF. The 3D co-culture model of pleura was incubated in SFM or stimulated with *S. aureus* in time-dependent response with 1 × 10^6^ CFU/mL bacteria concentration. The values are means 7 SE of four separate experiments. Statistical analysis was conducted using *t*-test (n = 7). Statistical significance ** *p* = 0.002, *** *p* = 0.003 and **** *p* < 0.0001 compared with control. (**b**) Permeability measurement using FITC-dextran assay. Permeability was measured by FITC-dextran (70 kDa) assay at 3 h, 6 h, 12 h, and 24 h incubation with *S. aureus*. The horizontal axis shows the fluorescence intensity of FITC-conjugated dextran leaking from the upper to the lower chambers of transwell membranes measured in each lower chamber 3 h after adding a molecular marker. The graph shows fluorescence intensity from the lower compartment. White, gray, and black bars indicate control (transwell, no cells seeded), 3D co-culture model of pleura not infected, and 3D tissue infected with 10^6^ CFU/mL of *S. aureus*, respectively. Error bars indicate SE (mean ± SEM; n = 8); *** *p* = 0.007, **** *p* < 0.0001 Student’s *t*-test. This figure is representative of three independent experiments. (**c**–**f**) Effect of *S. aureus* infection on junctional localization of VE-cadherin and ZO-1 proteins. Immunocytochemistry of VE-cadherin (c and d) and ZO-1 (**e**,**f**) on 3D co-culture model of pleura with or without *S. aureus* infection. Uninfected control 3D co-culture (**c**,**e**): VE-cadherin and ZO-1 staining clearly outline cell membranes of untreated HPMCs. At 3 h after incubation with *S. aureus* (**d**,**f**), the VE-cadherin and ZO-1 staining pattern is fragmented with disruption in VE-cadherin and ZO-1 continuity and incomplete appearance at the points of multiple cell contacts ((**d**,**f**) arrows). Controls where primary antibody has been omitted do not show any unspecific binding (data not included). Anti VE-cadherin (red), anti ZO-1 (orange), and DAPI (blue).

*S. aureus* already induced cytotoxic effects on cells 3 h after infection treated with 10^3^, 10^4^, 10^5^, or 10^6^ CFU/mL: 25%, 25%, 20%, and 34% of the total, with *p* < 0.05 for all time points compared to the control (Figure 5a). Increasing cytotoxic effects were observed for longer incubation times of 12 h (26, 42, 25, and 36%) and 24 h (41, 53, 51, and 90%), respectively (*p* < 0.0001 compared to vehicle control). Cell death was observed at all bacterial doses; however, more cytotoxic effects were detected in higher doses of bacteria (10^5^ and 10^6^ CFU/mL) 24 h post infection. In addition, the destruction of the 3D models after infection is caused by the translocation of bacteria across the cell layers (Figure 5b) and an increase in barrier permeability (see below, Figure 6b). 

Matching the VEGF-ELISA results, we measured the barrier permeability of the 3D co-culture model of pleura using an FITC-dextran assay. After the maturation time (day 1), the permeability of the 3D model was measured to detect changes in permeability during infection. The 3D co-culture of pleura incubated with *S. aureus* exhibited increased permeability to FITC-dextran (70 kDa) compared with models incubated with vehicle (Figure 6b). We found that *S. aureus* infection time-dependently enhanced mesothelial permeability in the 3D co-culture model (decrease in resistance by *p* = 0.0007 and *p* < 0.0001 at 3 h, 12 h, and 24 h, respectively).

The increase in macromolecule passage was paralleled by alterations in the distribution of the tight and adherens junctions by *S. aureus* infection. VE-cadherin and ZO-1 were localized in the cell membrane in the intercellular border (Figure 6c,e). *S. aureus* (1 × 10^3^ CFU/mL) caused a progressive disturbance in the continuity of VE-cadherin (Figure 6d) and ZO-1 (Figure 6f) localization at the cellular borders. As soon as 3 h after infection, VE-cadherin and ZO-1 localization at the cellular junctions were characterized by a gap-like appearance at points of cellular contact.

### 3.4. Characterization of the Biofilm in the 3D Co-Culture Model of Pleura

**Figure 7:**
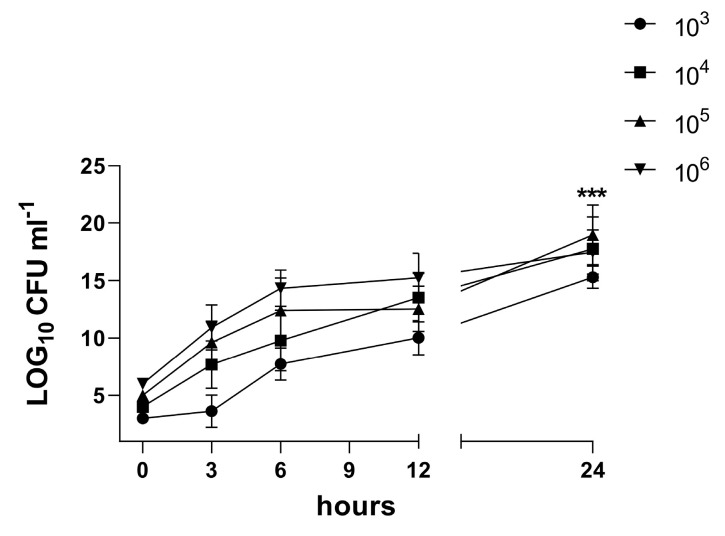
Biofilms of *S. aureus* (ATCC 49230) in 3D co-culture model monitored over 24 h. *S. aureus* colony-forming units (CFUs) within target sites of infection after inoculation over time. The 3D co-culture model was inoculated with *S. aureus*: ● = 10^3^ CFU/mL, ■ = 10^4^ CFU/ml, ▲ = 10^5^ CFU/ml, ▼ = 10^6^ CFU/ml. Bacterial burden was measured after 3 h, 6 h, 12 h, and 24 h. P values represent results for ANOVA test comparing CFUs at 0 and 24 h (*** *p* = 0.002). N = 6, error bars indicate SD.

We aimed to investigate the dynamics of host–pathogen interactions in our 3D model. Microscopy of H&E-stained sections of in vitro biofilms and samples from an infected 3D co-culture model revealed morphologically distinct bacteria residing close to each other (Figure 2a–o). Exposed sections were analyzed at multiple time points post infection for colonization and biofilm. Although the *S. aureus* culture triggered limited damage and single bacterial cells in the models in group B (10^3^ CFU/mL) or micro-colonies in group C (10^6^ CFU/mL) 3 h post infection, the 6 h incubation with bacteria resulted in the destruction of mesothelial connective layer components (Figure 2) and bacteria aggregates in group C (Table 1). At 12 h, the bacterial elements appeared tightly adhered in the submesothelial connective layer components and biofilm was formed. The 3D co-culture cells in both groups exposed for 24 h to the *S. aureus* culture demonstrated the destruction of mesothelial and submesothelial connective layers and the formation of a mature biofilm. The quantification and qualification of the biofilm were characterized using CFUs. The CFU enumeration of the bacteria per 3D model (Figure 7) shows a significant increase at 24 h compared to the time point 0 h after exposure (*p* = 0.002). At 24 h, maximum adhesion and internalization of the bacteria were observed in the case of all groups of inoculates (CFU = 10^3^–10^6^/mL).

## 4. Discussion

Infection of the mesothelium in visceral cavities such as pleural empyema is associated with high lethality. Pleural empyema, most commonly caused by pneumonia, cancer, or iatrogenic procedures, is a severe condition with frequent need for surgical intervention [32,33,34,35]. One of the main hurdles in the pathogenesis of pleural infection research has been the lack of an in vitro model that resembles human disease.

Rodent animal models do not reflect the human-specific reaction to pathogens. Thus, in this study, we investigated our recently established 3D co-culture model of human pleura [25] after infection with *S. aureus*. We aimed to analyze the site of infection and biofilm development. This model (Figure 1) allows examining different aspects of mesothelium infection simultaneously: bacterial attachment, invasion of the mesothelial barrier and transmigration through cell layers, stimulation of inflammatory responses from the host side, and formation of the mature bacterial biofilm by *S. aureus*. Here, we report about the dynamics of the *S. aureus* infection and its influence on HPMC in our 3D organotypic co-culture model.

Bacterial infection enormously influences the cellular communication, barrier properties, and cytokine production of mesothelial cells. PMCs are the first to come in contact with pathogens and their virulence. Therefore, mesothelial cells are strongly influenced by toxins and attachment molecules of bacteria, which enable loculation and septation in the pleural space and finally an injury of the pleural barrier with the development of parapneumonic effusions and pleural fibrosis [36].

We performed in vitro infection studies with different initial amounts of CFU/mL. Up to 12 h post infection we detected dissemination of bacteria throughout the complete 3D model and the induction of severe injury, disruption of mesothelial and submesothelial connective layers, and capsule formation (Figure 2). The histological appearance of the infected 3D models was comparable to late-stage empyema in humans, characterized by cell death of PMCs and desquamation of mesothelium [37,38]. Thus, the changes in cell architecture of the 3D co-culture model elicited by *S. aureus* were similar to in vivo data on empyema published by Mohammed et al. [31,39].

Matching our measurements of barrier permeability for macromolecules (FITC-dextran, 70 kDa) prior to and during infection to the VEGF-ELISA results, our model showed that *S. aureus* infection induced a rapid increase in permeability, starting at 3 h after infection. During later stages of infection (6 h, 12 h, and 24 h), the mesothelial and submesothelial connective layers showed evidence of severe disruption, leading to a massive decrease in FITC resistance. Due to the disruption of mesothelial integrity by toxins of *staphylococci*, our model did not sustain any barrier properties 6 h post exposure. This is supported by a reduction in transepithelial electrical resistance (TEER) and an increase in FITC-dextran permeability, comparable to data of previous studies demonstrating the effect of *S. aureus* on mucosa epithelium and human mesothelial cell monolayers [40,41,42,43].

*S. aureus* expresses an arsenal of virulence factors enabling it to promote colonization and invasion [8,44]. Secreted adhesins, such as cell wall lipoteichoic acid and teichoic acid, allow the pathogen to bind to membranes of human mesothelial cells, leading to denudation of the pleural surface and transmigration of pathogens throughout [37,38,43,45]. To address the question of how bacteria enter the pleural space during the development of empyema, we examined the transmigration of the pathogen. In concordance with published studies, we found that *S. aureus* can easily transmigrate across the HPMC barrier as early as 3 h after exposure. Pleural infection developed rapidly, with the majority of bacteria on the basal side of our 3D co-culture of pleura by 12 and 24 h post infection. Therefore, the cellular pathologies of our 3D co-culture model of pleura caused by *S. aureus* infection mimic the in vivo situation very well: HPMCs and the connective layer underneath did not act as a physical barrier against *S. aureus*.

It is well established that patients with pleural empyema caused by bacteria [31] and preclinical mouse models of empyema [46] show a presence of bacteria in the pleural cavity, an increase in pleural permeability, and a defective tight junction (TJ). Transmigration of *S. aureus* across the human tissue is triggered by the virulence factor SpA of *S. aureus,* which stimulates the activation of calpain. Calpain cleaves the junctional proteins, such as ZO-1, facilitating bacterial passage through the cell–cell junctions [47]. *S. aureus* virulence factors induced alteration of the HPMC TJ barrier (E-cadherin and ZO-1) in our 3D co-culture model of pleura (Figure 6c–f) and could be an important contributing factor in addition to tissue destruction by bacteria in the permeability defect (Figure 6b).

*S. aureus* infection causes a permeability change and barrier abnormality and enhances protein permeability in the pleural mesothelium [31,48]. VEGF is a key inducer of capillary permeability in acute and chronic diseases [31,49,50]. Its expression is upregulated in HPMCs activated by inflammation, thus affecting adherent junction proteins, such as E-cadherin and ZO-1, and leading to the formation of a pleural effusion [31,49,50]. In the pleural fluid of patients with inflammatory and malignant pleural effusion, VEGF is produced in large amounts [31,49,51]. PMCs have been shown to produce VEGF in response to infections by in vivo and in vitro studies [52]. In accordance with these findings, HPMCs in our 3D co-culture model of pleura released VEGF in a time-dependent manner when infected with *S. aureus* (Figure 6a). The results of the VEGF infection response presented here indicate that our 3D co-culture model is capable of secreting in vivo like levels of VEGF, in contrast to 2D models [31].

However, it is still unknown which of *S. aureus*’ virulence factors facilitate the translocation of bacteria across mesothelial cells and invasion into the bloodstream, thus causing sepsis, the most common cause of death in hospitals. Further studies are needed to identify the underlying mechanism, revealing a possible future target in empyema therapy.

It has been reported that bacteria trigger apoptosis and the disruption of mesothelial integrity [31,39]. Our data indicate that cell death was preceded by the activation of c-Jun in HPMCs during the initial phase of infection (Figure 4). C-Jun activity triggers apoptosis response in different cell types, including lung epithelial cells, fibroblasts, neural cells, keratinocytes, and melanocytes, among others [28,29,53,54,55,56]. C-Jun acts as an early response gene and induces the proapoptotic genes Bad and Bak in PMCs. Existing published models of HPMCs infected with *S. aureus* describe an increase in c-Jun gene expression, subsequent DNA fragmentation, and cytochrome-c release, causing the mesothelial cells to undergo apoptosis [43]. As shown in the present study, the effect of *S. aureus* infection on the 3D co-culture model of the pleura required 3 h for the first changes to occur. Increasing cytotoxic effects were observed for longer incubation times of 12 and 24 h (Figure 5a). These findings indicate that infection with *S. aureus* significantly induces VEGF expression and causes ultrastructural alteration of the mesothelial cells, which alters mesothelial permeability by affection of the junction proteins, such as VE-cadherin and ZO-1, and increased cell death.

Biofilm formation is one of *S. aureus*’ various virulence factors, but the mechanisms underlying the clinically relevant damage of pleural tissue remain unexplained. The dynamics of the biofilm infection and its influence on human cells was analyzed within our 3D co-culture model of pleura. Microscopy of in vitro biofilms and samples from the infected 3D co-culture model revealed morphologically distinct bacteria residing in close proximity to each other (Figure 2a–o). We showed that *S. aureus* strain ATCC 49230 is well able to form biofilms, perhaps reflecting the general biofilm lifestyle of *staphylococci* that colonize human body cavities during infections. Biofilm in the 3D co-culture model of pleura developed rapidly, with high numbers of bacteria and the formation of mature biofilm within 12 h post bacteria application.

Our model has the capacity to provide useful information on bacterial morphogenesis and survival, biofilm formation and pathology, as well as host–pathogen interactions in a physiologically relevant environment. We are convinced that this 3D organotypic co-culture model of pleura can potentially be used to demonstrate the effect of antibacterial agents, e.g., antibiotics, on the bacterial load on the mesothelium in patients in contrast to classical biofilm tests in cultures on glass or polystyrene [57]. It might add complexity and clinical relevance to the testing of antiseptics prior to clinical trials.

Two-dimensional monolayer cultures are narrowed to a single cell layer by limitation of cell nourishment [11]. In contrast, our 3D model allows for layering cells on top of each other by providing nourishment from the basal as well as the apical side. This also enables us to include two cell types in our model, a further advantage over monolayers by increasing complexity. While multilayered cell sheets also contain multiple cell types, only a single medium can be used in culture. Our methodology offers the advantageous possibility of using two different media and manipulating the ratio of cell types via changes in media.

Spheroid models also comprise multiple cell types. In contrast to our 3D model, they have the advantage of their extracellular matrix being produced by the cultured cells themselves [13]. Since cells in our model are dependent on cultivation in a collagen gel layer obtained from rat tails as an exogenous matrix, it is not entirely human-derived. For the purpose of this study, we opted for a 3D co-culture nonetheless, because its layered architecture represents the barrier layer function of pleura more accurately than the cell clusters of spheroids.

In the presented experiments, we cultivated the 3D models in 12-well plates, not allowing high-throughput testing such as in spheroid models, which are regularly produced in 96-well plates [13]. By using larger well plates and more inserts, testing can easily be upscaled.

Organ-on-a-chip microfluidic devices are a highly interesting combination of cell cultures and hollow devices enabling fluid flow in varying dynamics [58]. They enhance static cell culture models by mimicking the dynamic characteristics of, e.g., blood flow or breathing motions [58]. While they are a useful addition to 3D cell cultures by taking models one step closer to complex physiological conditions, they do not enable high-throughput testing. As of today, the materials used are rather expensive and non-disposable. Insufficient cleansing between two uses can taint complete experimental setups by contamination. In our 3D co-culture, risk of contamination is avoided by using disposable materials. This promising technique has already been used to produce several lung-on-chip models consisting of endothelial and lung cells type I and II at an air–liquid interface [59]. To our knowledge, no microfluidic device platform cultured with HPFs and HPMCs, thus representing pleura, exists.

One major limitation of our 3D model of pleura is the current restriction to only two cell types co-cultured (HPMCs and HPFs). In particular, the lack of innate immune cells delimitates the validity in inflammation studies. Future work will focus on improving the 3D co-culture via the integration of neutrophils, which will be key in future studies on infection and inflammation of mesothelial tissue.

In our investigations, the cell cultures were demonstrably viable for six days, offering only a short time frame for experiments. Three-dimensional models of other tissues, such as skin, have been successfully cultivated for longer periods [60], for up to thirty days in our experience. Thus, further improvement of the cultivation process can potentially achieve long-term viability.

Apart from studies on biofilm, our experimental in vitro setup also bears potential for investigations on lineage commitment, tissue regeneration, and wound healing. It could potentially also be used for the evaluation of new immunotherapeutic approaches as well as for pharmacological and toxicological studies, reducing the need for animal experiments. Among its further advantages are quick establishment, reproducibility of results, inexpensiveness, and sound correlation with in vivo clinical studies.

In conclusion, we were able to recreate a physiologically relevant microenvironment allowing co-cultured human fibroblasts and pleural mesothelial cells to show specific cytokine responses to pathogens on in vivo levels, resulting in a loss of permeability and cell death comparable to infected human pleura.

Thus, our non-animal, 3D organotypic in vitro co-culture model of human pleura offers biomimetic, clinically relevant conditions for a wide array of investigations.

## Figures and Tables

**Table 1:** Temporal development of biofilm on the 3D co-culture model of pleura.

Incubation Period (Hours)	Group B: *S. aureus* (1 × 10^3^ CFU/mL)	Group C: *S. aureus* (1 × 10^6^ CFU/mL)
**0**	no bacteria	no bacteria
**3**	single bacterial cells	bacterial micro-colonies
**6**	bacterial micro-colonies	bacterial aggregates
**12**	bacterial aggregates in tissue, biofilm	mature biofilm
**24**	mature biofilm	mature biofilm

## Data Availability

The data presented in this study are openly available in ORCID at https://doi.org/10.5281/zenodo.7732909.
